# Gelsemium

**Published:** 1867-12

**Authors:** D. L. Phares

**Affiliations:** Woodville, Minn.


### GELSEMIUM.

By D. L. Phares, M.D., Woodville, Minn.

In an article on this subject, published in the Journal some weeks ago, Dr. Jonathan W. Brooks takes to task the venerable and learned Prof. Wood, for inaccuracy in giving credit in "the article Gelseminum, or Yellow Jasmine, at pages 409 and 410 of the United States Dispensatory, twelfth edition," etc.

In the first place, Dr. B. does violence to the orthography of Prof. Wood, who, in accordance with highest authority and best usage, writes gelsemium, (an ancient name of the Jasmine,) and not gelsemi-n-um, from the more recent Italian form.

In the second place, Dr. B. lays violent hands on the phraseology of Prof. Wood, who never wrote such an ill-constructed, ungrammatical, and illogical sentence as that enclosed in quotation marks, as if from the United States Dispensatory.

In the third place, in attempting to convict Prof. Wood of ignorance, by an apparently learned array of authors, extending from the first to the nineteenth century, inclusive, Dr. B. succeeds in making an astonishingly brilliant display of his own deficiency of knowledge of the subject under consideration. For the authors cited, (one, perhaps, excepted,) at the very places and under the very titles referred to by Dr. B., say not a word about the plant of which Prof. Wood writes, but speak of one totally different in almost every respect. The Gelsimium having never been found in any part of the world but America, till introduced thence into Europe about the middle of the seventeenth century, it is no less absurd to seek for it in the "Materia Medica" of the third Dioscorides, than in the "*Hypomnemata*," or the "*Politeia Lakedaimonion*" of the second of that name, for a mention of the Constitution of Illinois; in the "*Epigrams*" of the second Dioscorides, for a notice of the literary character of the CHICAGO MEDICAL JOURNAL; in Aristotle's treatises on "*Sounds*" and "*Colors*," for the origin of Morse's Telegraph, or Photography; in Pliny's "Natural History of Animals," for an allusion to the American Opossum; or in Ptolemy's "*Geographike Aphegesis*," for the topography of Chicago. All would be equally relevant, pertinent, and satisfactory, being similar anachronisms.

On the other hand, the Jasminum, of which there many species, was known from a very early period in most countries of Europe, Asia, and Africa. It is the plant mentioned by Dioscorides and the others. It is the same that is named in "the article Jasminum officinale, by Charles Linnæus, 1743," as cited by Dr. B. The name is from the Arabic *ysmyn*, jasmine; though Linnæus, whose imagination was very lively, obtained a fancied etymology from the Greek *ia*, a violet, and *osme*, a smell. If Dr. B. will turn to pp. 1568–9 of the U.S. Dispensatory, he will find what Prof. Wood really does say of this plant, now, and for ages past, a favorite in the flower garden and conservatory, being the common white jessamine. The J.

hirsutum, more rare and esteemed, expands its myriads of delicate flowers only at night, in this latitude, lading the air, for a great distance around, with its delicious fragrance. But the most highly prized species, is the J. Sambac, a specimen of which, at the close of the seventeenth century, attracted so much attention in Hampton Court garden. When that was destroyed, this species was for a long time known in Europe only in the garden of the Grand Duke of Tuscany, at Pisa, where the plant was placed under guard, that no cuttings might be purloined. But I must forbear saying a tithe of what might be said with interest of this and other species of this very interesting genus.

Dr. B. will find the gelsemium (of which but one species is known) in the "Linnæan System," as well as in the earlier writers on the "Natural System," under the name *Bignonia sempervirens.* But however designated, the gelsemium and jasminum belong to totally different species, genera, families, tribes, orders, and classes; and differ no less in their properties, being no more akin than the raspberry and cockle-burr. All Dr. B.'s other remarks, arising from a misapprehension, are without a shadow of foundation, irrelevant, and require no further notice. He has been misled, evidently, by similarity of names, as we sometimes see one, misled by a name, direct his treatment to that, instead of the special morbid conditions of his patient.

All that Prof. Wood affirms of the medicinal properties of the gelsemium, and even much more, is true, and well known to many able practitioners of large experience in its use.

I close with Dr. B.'s closing words:—"The necessity of thus noticing" his "article is to be regretted."

